# *TP53* signature predicts pathological complete response after neoadjuvant chemotherapy for breast cancer: Observational and confirmational study using prospective study cohorts

**DOI:** 10.1016/j.tranon.2024.102060

**Published:** 2024-07-24

**Authors:** Shin Takahashi, Nobuaki Sato, Kouji Kaneko, Norikazu Masuda, Masaaki Kawai, Hisashi Hirakawa, Tadashi Nomizu, Hiroji Iwata, Ai Ueda, Takashi Ishikawa, Hiroko Bando, Yuka Inoue, Takayuki Ueno, Shinji Ohno, Makoto Kubo, Hideko Yamauchi, Masahiro Okamoto, Eriko Tokunaga, Shunji Kamigaki, Kenjiro Aogi, Hideaki Komatsu, Masahiro Kitada, Yasuaki Uemoto, Tatsuya Toyama, Yutaka Yamamoto, Toshinari Yamashita, Takehiro Yanagawa, Hiroko Yamashita, Yoshiaki Matsumoto, Masakazu Toi, Minoru Miyashita, Takanori Ishida, Fumiyoshi Fujishima, Satoko Sato, Takuhiro Yamaguchi, Fumiaki Takahashi, Chikashi Ishioka

**Affiliations:** a Department of Medical Oncology, Tohoku University Hospital, Sendai, Japan; b Department of Clinical Oncology, Tohoku University Graduate School of Medicine, Sendai, Japan; c Department of Breast Oncology, Niigata Cancer Center Hospital, Niigata, Japan; d Department of Breast and Endocrine Surgery, Nagoya University Graduate School of Medicine, Nagoya, Japan; e Department of Surgery, Breast Oncology, National Hospital Organization Osaka National Hospital, Osaka, Japan; f Department of Breast Surgery, Miyagi Cancer Center Hospital, Miyagi, Japan; g Department of Surgery I, Yamagata University Graduate School of Medical Science, Yamagata, Japan; h Department of Surgery, Tohoku Kosai Hospital, Sendai, Japan; i Department of Surgery, Hoshi General Hospital, Fukushima, Japan; j Department of Breast Oncology, Aichi Cancer Center Hospital, Nagoya, Japan; k Department of Breast Oncology and Surgery, Tokyo Medical University Hospital, Tokyo, Japan; l Breast and Endocrine Surgery, Institute of Medicine, University of Tsukuba, Ibaraki, Japan; m Breast Oncology Center, The Cancer Institute Hospital of Japanese Foundation for Cancer Research, Tokyo, Japan; n Department of Breast Surgical Oncology, Kyushu University Hospital, Kyushu University, Fukuoka, Japan; o Department of Breast Surgical Oncology, St. Luke's International Hospital, Tokyo, Japan; p Department of Breast Oncology, National Hospital Organization Kyushu Cancer Center, Fukuoka, Japan; q Department of Surgery, Sakai Municipal Hospital, Sakai, Japan; r Department of Breast Oncology, National Hospital Organization Shikoku Cancer Center, Ehime, Japan; s Department of Surgery, Iwate Medical University School of Medicine, Shiwa, Japan; t Breast Disease Center, Asahikawa Medical University Hospital, Asahikawa, Japan; u Department of Breast Surgery, Nagoya City University Graduate School of Medical Sciences, Nagoya, Japan; v Department of Breast and Endocrine Surgery, Kumamoto University Hospital, Kumamoto, Japan; w Department of Breast Surgery and Oncology, Kanagawa Cancer Center, Yokohama, Japan; x Department of Breast Surgery, Kansai Rosai Hospital, Amagasaki, Japan; y Department of Breast Surgery, Hokkaido University Hospital, Sapporo, Japan; z Breast Cancer Unit, Kyoto University Hospital, Graduate School of Medicine, Kyoto, Japan; aa Department of Breast and Endocrine Surgical Oncology, Tohoku University Graduate School of Medicine, Sendai, Japan; bb Department of Pathology, Tohoku University Hospital, Sendai, Japan; cc Division of Biostatistics, Tohoku University Graduate School of Medicine, Sendai, Japan; dd Division of Medical Engineering, Department of Information Science, Iwate Medical University, Yahaba, Japan

**Keywords:** Breast cancer, *TP53*, Neoadjuvant chemotherapy, Pathological complete response, Predictive factor

## Abstract

The *TP53* signature is considered a predictor of neoadjuvant chemotherapy (NAC) response and prognostic factor in breast cancer. The objective of this study was to confirm *TP53* signature can predict pathological complete response (pCR) and prognosis in cohorts of breast cancer patients who received NAC in prospective studies.

Development cohorts (retrospective [*n* = 37] and prospective [*n* = 216] cohorts) and validation cohorts (NAC administered prospective study cohorts [*n* = 407] and retrospective perioperative chemotherapy (PC)-naïve, hormone receptor (HrR)-positive cohort [PC-naïve_HrR+ cohort] [*n* = 322]) were used. *TP53* signature diagnosis kit was developed using the development cohorts. *TP53* signature predictability for pCR and the relationship between recurrence-free survival (RFS), overall survival (OS), and the *TP53* signature were analyzed.

The pCR rate of the mutant (mt) signature group was significantly higher than that of the wild-type (wt) signature group (odds ratio, 5.599; 95 % confidence interval = 1.876–16.705; *P* = 0.0008). The comparison of the RFS and OS between the HrR+ and HER2− subgroup of the NAC cohort and of the PC-naïve_HrR+ cohort indicated that the RFS and OS benefit of NAC was greater in the mt signature group than in the wt signature group. From post hoc analyses, the RFS and OS benefit from adding capecitabine to FEC+T as NAC might be observed only in the mt signature group.

The *TP53* signature can predict the pCR after NAC, and the RFS and OS benefit from NAC may be greater in the mt signature group than in the wt signature group.

## Highlights

• *TP53* signature can predict the pathological complete response in breast cancer.
• *TP53* signature is a predictor for survival benefit from neoadjuvant chemotherapy.
• Benefit from chemotherapy is grater in mutant than in wild-type signature group.

## Introduction

Perioperative chemotherapy (neoadjuvant chemotherapy [NAC] and adjuvant chemotherapy) plays an important role in the treatment of patients with breast cancer [1]. A meta-analysis by the Early Breast Cancer Trialists’ Collaborative Group (EBCTCG) has revealed that the prognosis of patients who underwent NAC was comparable with that of patients who underwent adjuvant chemotherapy [2]. Furthermore, the study reported a higher rate of breast-conserving surgery in patients who received NAC compared to those who received adjuvant chemotherapy. Another meta-analysis reported that patients who achieved pathological complete response (pCR) after NAC had a better prognosis than those who did not achieve pCR [3,4]. Additionally, studies have shown that the prognosis of patients who did not achieve pCR after NAC improved with the addition of adjuvant chemotherapy [5,6]. Recently, the efficacy of immune checkpoint inhibitors in NAC has been demonstrated in triple negative breast cancer (TNBC) [[7], [8], [9], [10]]. Furthermore, various factors have been reported to be associated with the therapeutic efficacy of immune checkpoint inhibitors [[11], [12], [13]]. Additionally, novel agents with high therapeutic efficacy have been developed and are expected to be useful in NAC [14,15]. Indeed, the importance of NAC in perioperative chemotherapy is perpetually increasing. However, no useful method for predicting pCR has been developed thus far.

The tumor suppressor gene *TP53* is one of the most frequently mutated genes in human malignancies [16,17]. Although the biological significance of *TP53* mutations has been reported to vary by cancer type [18], it is known to be a prognostic factor in breast cancer [19]. *TP53* signature is a gene expression profile that predicts the mutation status of the *TP53* gene [20]. The *TP53* signature comprises 24 upregulated and 9 downregulated genes in patients with *TP53* gene mutations. It serves as a prognostic factor; breast cancer patients with *TP53* wild-type (wt) signature have a better prognosis than those with mutant (mt) signature [[20], [21], [22], [23], [24]]. Moreover, *TP53* signature predicted response to chemotherapy; the pCR rate of breast cancer patients with *TP53* mt signature was significantly higher than that of breast cancer patients with *TP53* wt signature [[24], [25], [26]].

Notably, all the above mentioned studies were observational in design and used retrospective cohorts. To the best of our knowledge, no study has examined the clinical utility of the *TP53* signature using cohorts from prospective clinical studies.

The purpose of this study was to develop a *TP53* signature diagnostic system and to evaluate whether *TP53* signature could predict pCR and prognosis in cohorts of patients who received NAC in prospective clinical studies.

## Methods

### Study design

**Fig. 1:**
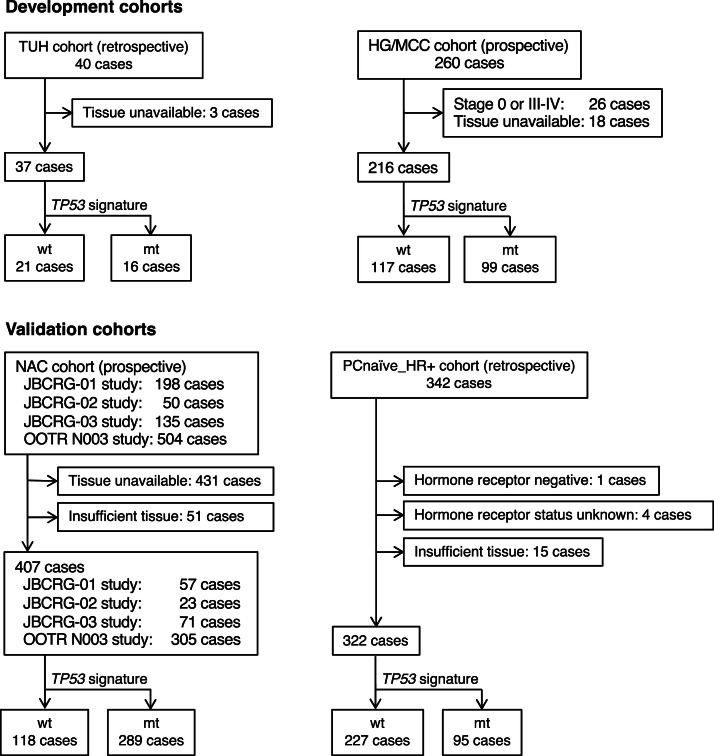
Consort diagram TUH, Tohoku University Hospital; HG/MCC, Hoshi General Hospital/Miyagi Cancer Center; JBCRG, Japan Breast Cancer Research Group; OOTR, Organization of Oncology and Translational Research; NAC, neoadjuvant chemotherapy; wt, wild-type signature, mt, mutant signature.

This study was an observational and confirmational cohort study,performed in accordance with a pre-defined protocol and statistical analysis plan. It utilized both development cohorts (Tohoku University [TUH] cohort and Hoshi General Hospital/Miyagi Cancer Center [HG/MCC] cohort) and validation cohorts (NAC cohort and perioperative chemotherapy [PC]-naïve, hormone receptor-positive breast cancer cohort) as detailed below (Fig. 1). Details of cohorts are described in the Supplemental information.

### The development cohorts

The Tohoku University Hospital (TUH) cohort (*n* = 37): A cohort of primary breast cancer patients who underwent surgery at TUH. *TP53* gene mutation data were obtained via direct sequencing in our previous study [20].

The Hoshi General Hospital/Miyagi Cancer Center (HG/MCC) cohort (*n* = 216): a cohort from a prospective observational study in primary breast cancer patients who received surgery between February 2008 and February 2014 [21,22].

The TUH cohort and HG/MCC cohort were used to develop the *TP53* signature diagnostic kit and determine the cut-off value for diagnosing *TP53* signature status. The HG/MCC cohort was used to evaluate the prognostic value of the *TP53* signature.

### The validation cohorts

NAC cohort (*n* = 407): Cohort from 5 prospective clinical studies (JBCRG-01, JBCRG-02, JBCRG-02′, JBCRG-03, and OOTR-N003) in which NAC was administered to primary breast cancer patients with cT1c-3 cN0 cM0 (>1 cm) / cT1–3 cN1 cM0 [[27], [28], [29], [30], [31]]. Patients whose specimens were available were included in the analysis.

Perioperative chemotherapy (PC)-naïve, hormone receptor-positive (positive for both or one of ER and PgR) breast cancer cohort (PC-naïve_HrR+ cohort) (*n* = 322): A retrospectively collected cohort of HrR+ breast cancer patients (cT1c-3 cN0 cM0 (>1 cm) / cT1–3 cN1 cM0; which is same as the patient selection criteria of the NAC cohort) who underwent surgery without PC between August 2005 and July 2009 for matching the enrollment period of the NAC cohort. To eliminate case selection bias, we enrolled up to 60 consecutive eligible patients per institution from August 1, 2005.

The NAC cohort was used to test whether the *TP53* signature diagnosis kit could predict pCR after NAC. The PC-naïve_HrR+ cohort was compared with the HrR+ subgroup of the NAC cohort to assess differences in the prognostic significance of NAC based on *TP53* signature status.

This study was designed in accordance with the Declaration of Helsinki (2013 revision) and the Japanese Guidelines for the Ethics of Clinical Research. It was reviewed and approved by the ethics committee of Tohoku University Hospital (Approved on June 25, 2019, Approval number: 15061) and each participating institution. As this was an observational study and did not involve invasive procedures, informed consent (including consent for publication) was obtained through the opt out method. To ensure participants had the opportunity to opt out, information related to this study was published on facility websites (https://sites.google.com/alpha.crieto.med.tohoku.ac.jp/koukai/). This study was registered in UMIN—CTR (http://www.umin.ac.jp/ctr/) (000037505).

### *TP53* signature diagnosis kit and method for diagnosis of *TP53* signature status

Details of the *TP53* signature diagnostic kit and diagnostic procedure are described in Supplemental information and Supplemental Table 1. The ratio of the sum of logarithmic expression levels of 18 upregulated genes to the sum of logarithmic expression levels of 9 downregulated genes was used to define the *TP53* signature score [21]. If the *TP53* signature score of a sample was equal to or greater than the cut-off value, the sample was labeled as having a *TP53* mt signature; otherwise, it was labeled as having a *TP53* wt signature. The optimal cut-off *TP53* signature score was determined using receiver operating characteristic (ROC) analysis.

### Specimens and RNA extraction

For the NAC cohort, formalin-fixed, paraffin-embedded (FFPE) specimens from biopsies performed before NAC were used. For the remaining three cohorts, either FFPE biopsy specimens or surgical specimens were used. Details are described in the Supplemental information.

### Clinicopathological factors

Details of collected clinicopathological background factors are described in the Supplemental information.

### Endpoints

The primary endpoint of the study was pCR, and the hypothesis being tested was that the rate of pCR in the *TP53* mt signature group would be higher than that in the wt signature group within the NAC cohort. The pCR was defined as quasi-pCR (QpCR), which refers to the absence of invasive residual disease in the breast, while allowing for non-invasive residual disease, a small amount of remaining invasive residual disease, and infiltrated lymph nodes [32]. The secondary endpoints were overall survival (OS) and recurrence-free survival (RFS).

### Statistical analyses

Statistical analysis was performed in accordance with a pre-defined statistical analysis plan.

SAS ver 9.4 R (SAS Institute, Cary, NC, USA) or JMP Pro 16.0.0 (SAS Institute Japan, Tokyo, Japan) was used for statistical analyses. The chi-squared test was used to analyze all patient background variables except age, while the Kruskal–Wallis test was used to analyze patient age. The Kaplan–Meier method was used to generate survival curves, and intergroup differences were compared using the log-rank test.

Logistic regression analyses were performed using the *TP53* signature type (mt or wt) as the outcome variable and ER, PgR, HER2, Ki-67 (continuous variable), age (continuous variable), menopausal status, T, N, and stage as covariates to calculate the propensity score. The propensity score was used to divide the cases into three equal-sized strata, and the pCR rates, 95 % confidence intervals (95 % CI) via the Clopper–Pearson method, and odds ratio (OR)s of the mt signature and wt signature groups were calculated. In addition, the Cochran–Mantel–Haenszel common OR and its CI were calculated, and the main null hypothesis that the common OR was 1 was tested using the Mantel–Haenszel test with a two-sided significance level of 5 %. The Breslow–Day test was used to evaluate the homogeneity of the ORs across different strata to ensure consistency in the effect of the *TP53* signature on pCR rates. Similar analyses were performed using ER and PgR as stratification factors.

A logistic regression model was used with pCR as the outcome variable and *TP53* signature type, ER, PgR, HER2, Ki-67, age, menopausal status, T, N, and stage as covariates to estimate the effect of the *TP53* signature type adjusted by covariates and its CI. *P* < 0.05 indicated statistical significance.

As a post-hoc analysis, we investigated the relationship between the effect of adding capecitabine to NAC and the *TP53* signature in the OOTR-N003 study cohort.

## Results

### Cut-off value for *TP53* signature diagnostic system

The *TP53* signature score for classifying cases into mt-like and wt-like breast cancer was determined to be 1.67 (area under the ROC curve = 0.97994) using ROC analysis. Details of the results are described in the Supplemental information, Supplemental Fig. 1 and Supplemental Table 2.

### Prognostic significance of the *TP53* signature diagnostic system in the HG/MCC cohort

Patient clinicopathological background factors of the HG/MCC cohort are presented in the Supplemental information (Supplemental Table 3). Details about comparisons of patient clinicopathological background factors are described in the Supplemental information. In all cases of the cohort, pStage I, pStage II, and ER-positive subgroups, the RFS of the *TP53* mt signature group was significantly shorter than that of the wt signature group (Supplemental Fig. 2A, B, C, and D). In the HER2-positive and triple negative breast cancer (TNBC) subgroups, no significant difference was noted in RFS between the two *TP53* signature groups; however, it is noteworthy that no patient in the wt-signature group experienced recurrence (Supplemental Fig. 2E and F).

### Validation cohort

In the NAC cohort and the PC-naïve_HrR+ cohort, 118 and 289 cases, and 227 and 95 cases had *TP53* wt signature and mt signature, respectively (Table 1). In both cohorts, the *TP53* mt signature group had significantly more ER-negative, PgR-negative, HER2-positive, higher grade, and higher Ki-67 cases than the wt signature group.

**Table 1:** Patient clinicopathological background of NAC and PC-naïve_HrR+ cohort disaggregated by *TP53* signature status.

	NAC cohort							PC-naïve_HrR+ cohort							
	All (*n* = 407)		wt signature (*n* = 118)		mt signature (*n* = 289)			All (*n* = 322)		wt signature (*n* = 227)		mt signature (*n* = 95)			
	n	%	n	%	n	%	P*	n	%	n	%	n	%	P*	P* NAC cohort vs. PC-naïve_HrR+ cohort
Age median (Range)	48 (24–70)		46 (30–68)		48 (24–70)		0.073	52 (27–69)		53 (29–69)		52 (27–69)		0.19	<0.0001
Menopausal status							0.26							1.00	<0.0001
Premenopausal	254	62.4	79	66.9	175	60.6		153	47.5	108	47.6	45	47.4		
Postmenopausal	153	37.6	39	33.1	114	39.4		169	52.5	119	52.4	50	52.6		
pT							0.22							0.7	<0.0001
T1	20	4.9	8	6.8	12	4.2		202	62.7	144	63.4	58	61.1		
T2	336	82.6	99	83.9	237	82.0		119	37.0	82	36.1	37	38.9		
T3	51	12.5	11	9.3	40	13.8		1	0.3	1	0.4	0	0.0		
pN							0.51							0.40	<0.0001
N0	213	52.3	65	55.1	148	51.2		292	90.7	208	91.6	84	88.4		
N1	194	47.7	53	44.9	140	48.4		30	9.3	19	8.4	11	11.6		
ER							<0.0001							0.30	<0.0001
positive	277	68.1	110	93.2	167	57.8		321	99.7	227	100.0	94	98.9		
negative	123	30.2	6	5.1	117	40.5		1	0.3	0	0.0	1	1.1		
NA	7	1.7	2	1.7	5	1.7		0	0.0	0	0.0	0	0.0		
PgR							<0.0001							0.0011	<0.0001
positive	219	53.8	96	81.4	123	42.6		271	84.2	201	88.5	70	73.7		
negative	178	43.7	20	16.9	158	54.7		51	15.8	26	11.5	25	26.3		
NA	10	2.5	2	1.7	8	2.8		0	0.0	0	0.0	0	0.0		
HER2							<0.0001							0.0009	<0.0001
positive	75	18.4	6	5.1	69	23.9		8	2.5	1	0.4	7	7.4		
negative	320	78.6	108	91.5	212	73.4		280	87.0	203	89.4	77	81.1		
NA	12	2.9	4	3.4	8	2.8		34	10.6	23	10.1	11	11.6		
Grade							<0.0001							<0.0001	<0.0001
1	64	15.7	45	38.1	19	6.6		205	63.7	176	77.5	29	30.5		
2	183	45.0	55	46.6	128	44.3		94	29.2	50	22.0	44	46.3		
3	121	29.7	7	5.9	114	39.4		23	7.1	1	0.4	22	23.2		
NA	39	9.6	11	9.3	28	9.7		0	0.0	0	0.0	0	0.0		
Ki-67							<0.0001							<0.0001	0.65
<10 %	239	58.7	87	73.7	152	52.6		178	55.3	152	67.0	26	27.4		
≧10%	163	40.0	27	22.9	136	47.1		129	40.1	61	26.9	68	71.6		
NA	5	1.2	4	3.4	1	0.3		15	4.7	14	6.2	1	1.1		

### Prediction of pCR by *TP53* signature

In the NAC cohort, ER, PgR, Ki-67, age, menopausal status, T, N, stage, and pCR data were available for 381 cases. The results of stratified analysis of the pCR rates of these cases using the propensity score as a stratification factor are shown in Table 2. The results of the Breslow–Day test suggested the absence of qualitative differences between the strata (*P* = 0.562). The Cochran–Mantel–Haenszel OR of the mt signature group to the wt signature group was 5.599 (95 % CI = 1.876–16.705), and the pCR rate of the mt signature group was significantly higher than that of the wt signature group (*P* = 0.0008).

**Table 2:** Stratified analysis of pCR rate by the propensity score.

Strata	*TP53* signature	n	pCR (%; 95 % CI)	Non-pCR	OR (95 % CI)	*P*
1	wt	66	2 (3.0; 0.2–10.9)	64	1	
	mt	62	5 (8.1; 2.7–17.8)	57	2.807 (0.524–15.035)	
2	wt	41	2 (4.9; 0.6–16.5)	39	1	
	mt	85	22 (27.1; 17.0–36.5)	63	6.810 (1.517–30.567)	
3	wt	3	0 (0.0; 0.0–70.8)	3	−	
	mt	124	54 (43.5; 34.7–52.7)	70	−	
Cochran-Mantel-Haenszel test					5.599(1.876–16.705)	0.0008
Breslow-Day test						0.562

Abbreviations: pCR, pathological complete response; OR, odds ratio; CI, confidence interval; wt, wild-type; mt, mutant.

The stratified analysis of the pCR rates with the ER and PgR as stratification factors (*n* = 391) also showed that the Cochran–Mantel–Haenszel OR of the mt signature group to the wt signature group was 4.789 (95 % CI = 1.811–12.665), and the pCR rate of the mt signature group was significantly higher than that of the wt signature group (*P* = 0.0007) (Supplemental Table 4).

The logistic regression analysis with the pCR rate revealed that the adjusted OR of the mt signature group versus the wt signature group was 6.463 (95 % CI = 2.162–19.324, *P* = 0.0008) (Supplemental Table 5).

### The prognostic significance of the *TP53* signature for prognosis in the validation cohort

**Fig. 2:**
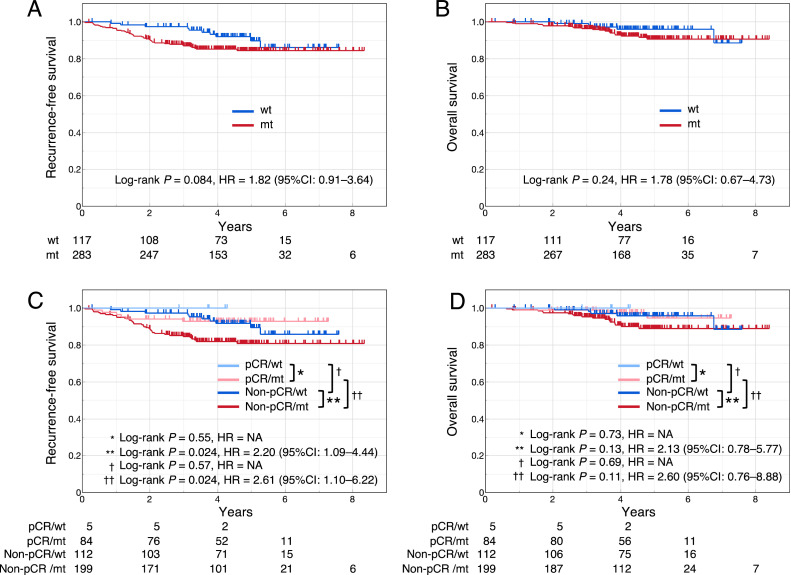
RFS and OS of NAC cohort RFS (A) and OS (B) stratified according to *TP53* signature status; RFS (C) and OS (D) stratified according to pCR status and *TP53* signature status. RFS, recurrence-free survival; OS, overall survival; NAC, neoadjuvant chemotherapy; pCR, pathological complete response; wt, wild-type signature, mt, mutant signature; HR, hazard ratio; CI: confidence interval; NA, not available.

The prognostic significance of the *TP53* signature for prognosis was examined in the NAC cohort. Data on RFS and OS were missing for 1 case in the wt signature group and 6 cases in the mt signature group. In all cases of the cohort, the RFS of the mt signature group tended to be shorter than that of the wt signature group, although the difference was not significant (log-rank *P* = 0.084; hazard ratio [HR] = 1.82 [95 % CI = 0.91–3.64]) (Fig. 2A). No significant intergroup difference was observed in OS (log-rank *P* = 0.24, HR = 1.78 [95 % CI = 0.67–4.73]) (Fig. 2B).

In the HrR+, TNBC, and HER2+ subgroups, no significant differences in RFS or OS were noted between the mt and wt signature groups (Supplemental Fig. 3A–F).

In cases with pCR, no significant difference in RFS was observed between the wt and mt signature groups (log-rank *P* = 0.55, HR = not available [NA]) (Fig. 2C). In cases with non-pCR, the RFS of the mt signature group was significantly shorter than that of the wt signature group (log-rank *P* = 0.024; HR = 2.20 [95 % CI = 1.09–4.44]). Despite the absence of a significant difference in RFS between cases with pCR and non-pCR in the wt signature group (log-rank *P* = 0.57, HR = not available [NA]), RFS of the non-pCR group was significantly worse than that of the pCR group in the mt signature group (log-rank *P* = 0.024; HR = 2.61 [95 % CI = 1.10–6.22]). No significant differences were noted in OS between any of the two groups (Fig. 2D).

### Significance of *TP53* signature in HrR+ and HER2− breast cancer

To investigate the relationship between the *TP53* signature and the clinical outcomes after NAC in HrR+ and HER2− (HrR+/HER2−) breast cancer, we compared RFS and OS between patients with HrR+/HER2− in the NAC cohort [NAC (HrR+/HER2−)] and patients with HER2− in the PC-naïve_HrR+ cohort [PC-naïve (HrR+/HER2−)]. Comparisons of patient clinicopathological background factors disaggregated by *TP53* signature status in NAC (HrR+/HER2−) cohort and PC-naïve (HrR+/HER2−) cohort are shown in Supplemental Table 6. The NAC (HrR+/HER2−) group had a higher proportion of patients with poor prognostic factors than in the PC-naïve (HrR+/HER2−) group. Further details about these comparisons are described in the Supplemental information.

**Fig. 3:**
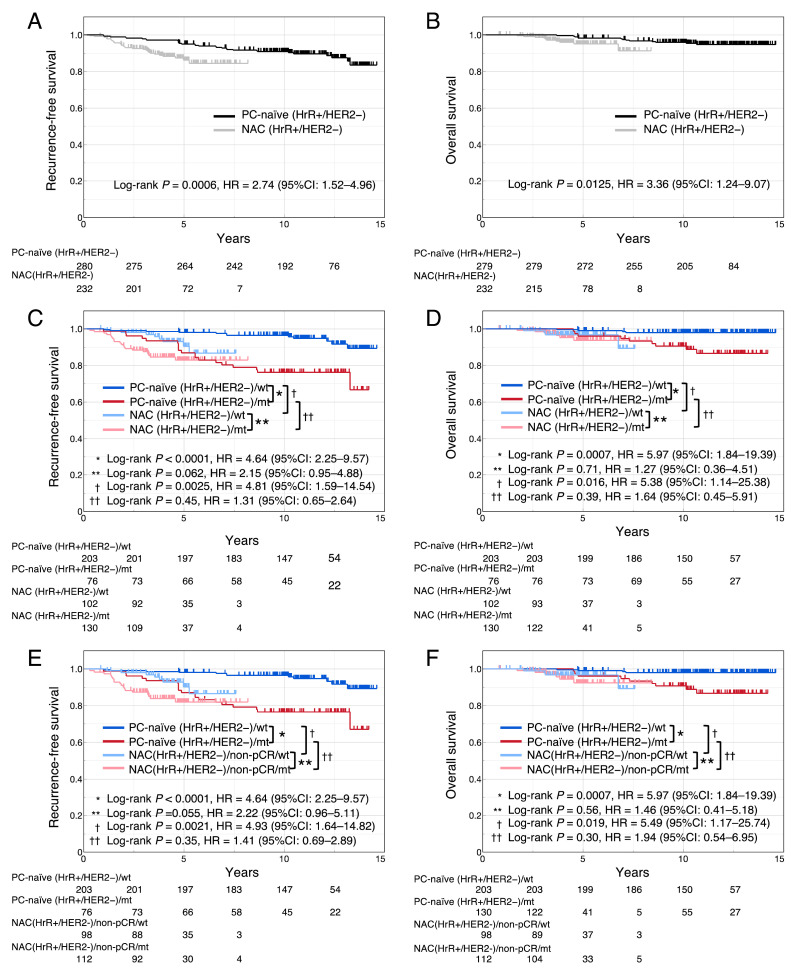
RFS and OS of PC-naïve (HrR+/HER2−) and NAC (HrR+/HER2−) cohort RFS (A) and OS (B) stratified according to cohort; RFS (C) and OS (D) stratified according to cohort and *TP53* signature status. RFS (E) and OS (F) stratified according to cohort and *TP53* signature status, excluding cases with pCR from the NAC (HrR+/HER2−) cohort. RFS, recurrence-free survival; OS, overall survival; PC-naïve, perioperative chemotherapy-naïve; HrR, hormone receptor; HER2, human epidermal growth factor receptor type 2; NAC, neoadjuvant chemotherapy; pCR, pathological complete response; wt, wild-type signature, mt, mutant signature; HR, hazard ratio; CI: confidence interval.

Data on RFS and OS were missing for 4 cases in the NAC (HrR+/HER2−) cohort. RFS and OS of the NAC (HrR+/HER2−) cohort were significantly shorter than those of the PC-naïve (HrR+/HER2−) cohort (RFS, log-rank *P* = 0.0006, HR = 2.74 [95 % CI = 1.52–4.96]; OS, log-rank *P* = 0.0125, HR = 3.36 [95 % CI = 1.24–9.07]) (Fig. 3A and B).

The comparison of the RFS and OS between NAC (HrR+/HER2−) cohort and the PC-naïve (HrR+/HER2−) cohort by dividing them into wt and mt signature groups are shown in Fig. 3C and D. In the PC-naïve (HrR+/HER2−) cohort, the RFS and OS of the mt signature group were significantly shorter than those of the wt signature group (RFS, log-rank *P* < 0.0001, HR = 4.64 [95 % CI = 2.25–9.57]; OS, *P* = 0.0007, HR = 5.97 [95 % CI = 1.84–19.39]). In contrast, in NAC (HrR+/HER2−) cohort, RFS and OS showed no significant differences between the mt and wt signature groups (RFS, log-rank *P* = 0.062, HR = 2.15 [95 % CI = 0.95–4.88]; OS, log-rank *P* = 0.71, HR = 1.27 [95 % CI = 0.36–4.51]). Between the wt signature groups in the two cohorts, RFS and OS of the NAC (HrR+/HER2−)/wt group were significantly shorter than those of the PC-naïve (HrR+/HER2−)/wt group (RFS, log-rank *P* = 0.0025, HR = 4.81 [95 % CI = 1.59–14.54); OS, log-rank *P* = 0.016, HR = 5.38 [95 % CI = 1.14–25.38]). However, for the mt signature groups in the two cohorts, there were no differences in RFS or OS between the NAC (HrR+/HER2−)/mt group and the PC-naïve (HrR+/HER2−)/mt group (RFS, log-rank *P* = 0.45, HR = 1.31 [95 % CI = 0.65–2.64]; OS, log-rank *P* = 0.39, HR = 1.64 [95 % CI = 0.45–5.91]). Furthermore, as a post hoc analysis, a similar analysis was performed excluding cases with pCR from the NAC cohort, demonstrating similar results to those described above (Fig. 3E and F).

### Differences in benefit from adding capecitabine to NAC between mt and wt signatures

The OOTR-N003 study was conducted to compare the efficacy of FEC + T (docetaxel) with FEC + TX (docetaxel + capecitabine) as NAC. As a post hoc analysis of the present study, we investigated whether there was any association between the *TP53* signature status and the RFS and OS of the two groups. The comparison of patient clinicopathological background factors (Supplemental Table 7) showed no significant intergroup differences except in terms of T stage. Details about these comparisons are described in the Supplemental information.

**Fig. 4:**
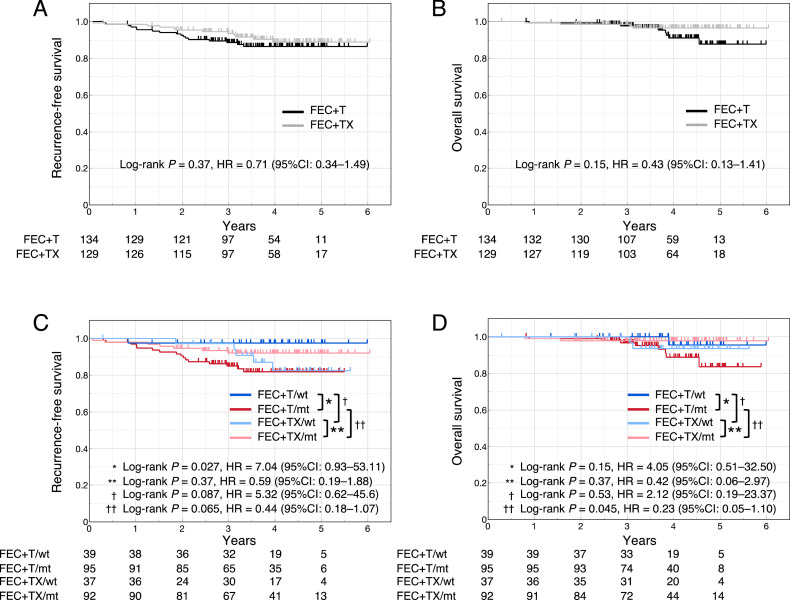
RFS and OS of the OOTR-N003 cohort RFS (A) and OS (B) stratified according to the treatment regimens. RFS (C) and OS (D) stratified according to the treatment regimens and *TP53* signature status. RFS, recurrence-free survival; OS, overall survival; FEC, fluorouracil, epirubicin, and cyclophosphamide; T, docetaxel; X, capecitabine; wt, wild-type signature, mt, mutant signature; HR, hazard ratio; CI: confidence interval.

Data on RFS and OS were missing for 1 case in each of the FEC+T and FEC+TX groups. There were no significant differences in RFS and OS between the FEC+T and FEC+TX groups (RFS, log-rank *P* = 0.37, HR = 0.71 [95 % CI = 0.34–1.49]; OS, log-rank *P* = 0.15, HR = 0.43 [95 % CI = 0.13–1.41]) (Fig. 4A and B). The RFS of the FEC+T/mt group was significantly shorter than that of the FEC+T/wt group (log-rank *P* = 0.027, HR = 7.04 [95 % CI = 0.93–53.11]), whereas RFS did not differ significantly between the FEC+TX/mt and FEC+TX/wt groups (log-rank *P* = 0.37, HR = 0.59 [95 % CI = 0.19–1.88]) (Fig. 4C). Moreover, the RFS of the FEC+TX/wt group tended to be shorter than that of the FEC+T/wt group (log-rank *P* = 0.087, HR = 5.32 [95 % CI = 0.62–45.6]). Conversely, the RFS of the FEC+TX/mt group tended to be longer than that of the FEC+T/mt group (log-rank *P* = 0.065, HR = 0.44 [95 % CI = 0.18–1.07]) (Fig. 4C). The OS of the FEC+TX/mt group was significantly better than that of the FEC+T/mt group (log-rank *P* = 0.045, HR = 0.23 [95 % CI = 0.05–1.10]) (Fig. 4D). In contrast, we noted no significant differences in OS between the following groups: FEC+T/mt and FEC+T/wt, FEC+TX/mt and FEC+TX/wt, and FEC+T/wt and FEC+TX/wt.

## Discussion

The aim of this observational study was to assess and confirm the predictive value of the *TP53* signature for achieving pCR in patients receiving NAC, based on data obtained from cohorts enrolled in prospective clinical studies. The OR of the mt group relative to the wt group was 5.599 (95 % CI = 1.876–16.705, *P* = 0.0008), demonstrating that the pCR rate of the mt group was significantly higher than that of the wt group. Logistic regression analysis further confirmed that the *TP53* signature served as the strongest and most independent predictor of pCR, even after accounting for other clinicopathological factors. This study represents the first confirmation of the previous reports in cohorts derived from prospective clinical studies.

In the entire NAC cohort, although there was no significant difference in RFS between the mt and wt signature groups (Fig. 2A), in non-pCR cases, RFS in the mt signature group was significantly worse than in the wt signature group (Fig. 2C). Therefore, one reason for these results may be that the pCR rate in the mt signature group was significantly higher than that in the wt signature group (Table 2).

The fact that patients achieving pCR after NAC generally have a more favorable prognosis compared to those with non-pCR is well known [3,4]. However, there is a possibility that NAC contributes to an improved prognosis even in cases with non-pCR. Furthermore, the prognostic impact of NAC may differ between wt and mt signature groups. To test this hypothesis, we focused on analyzing the association between *TP53* signature and prognosis in the NAC (HrR+/HER2−) and PC-naïve (HrR+/HER2−) cohorts. The reason for limiting the comparison to HrR+/HER2− cases was that the majority of HER2-positive and TNBC patients underwent perioperative chemotherapy, and it was difficult to collect a sufficient number of HER2-positive and TNBC patients who did not receive perioperative chemotherapy. When comparing the clinicopathological background factors between cohorts, the NAC (HrR+/HER2−) cohort had a higher prevalence of poor prognostic factors compared with the PC-naive (HrR+/HER2−) cohort (Supplemental Table 6). Therefore, the former cohort exhibited a poorer prognosis than the latter cohort (Fig. 3A and B). Although the RFS and OS of the mt signature group were poorer than those of the wt signature group in the PC-naïve (HrR+/HER2−) cohort, no differences in prognosis were noted between the two groups in the NAC (HrR+/HER2−) cohort (Fig. 3C and D). If the prognostic benefit of NAC is equivalent for the wt and mt signature groups, then the HRs for the wt and mt signature groups in the PC-naïve (HrR+/HER2−) cohort should be comparable to those in the NAC (HrR+/HER 2−) cohort. Thus, this result suggests that the prognostic benefit of NAC is greater in the mt signature group than in the wt signature group. In addition, while the RFS and OS of NAC (HrR+/HER2−)/wt were significantly worse than those of PC-naïve (HrR+/HER2−)/wt, there was no difference in RFS and OS between NAC (HrR+/HER2−)/mt and PC-naïve (HrR+/HER2−)/mt. In both wt and mt signature groups, the NAC (HrR+/HER 2−) cohort had significantly more patients with poor prognostic clinicopathological factors than the PC-naïve (HrR+/HER2−) cohort (Supplemental Table 6). Therefore, if the prognostic benefit of NAC is equivalent in the wt and mt signature groups, the HR between NAC (HrR+/HER2−) and PC-naïve (HrR+/HER2−) cohorts in the *TP53* signature wt group should be comparable to that in the mt group. Therefore, this result also suggests that the prognostic benefit of NAC is greater in the mt signature group, whereas it is limited in the wt signature group. Since these results could be attributed to the fact that the mt signature group contained more cases with pCR after NAC, the same analysis was performed excluding cases with pCR from the NAC (HrR+/HER2−) cohort. The results were similar to the previous results, even in non-pCR cases (Fig. 3E and F); thus, the prognostic benefit of NAC was greater in the mt signature group than in the wt signature group in non-pCR cases.

NAC was more beneficial in the mt signature group than in the wt signature group, suggesting that more intensive NAC may be more beneficial in the former. To examine this hypothesis, we examined the relationship between *TP53* signature status and prognosis using the OOTR-N003 cohort, which compared FEC+T and FEC+TX as NAC regimens [31] (Fig. 4). In the FEC+T group, the RFS of the mt signature group was significantly shorter than that of the wt signature group, whereas no significant difference in RFS was observed between the two groups in the FEC+TX group. In the wt signature groups, the RFS of the FEC+TX group showed a tendency to be worse than that of the FEC+T group [log-rank *P* = 0.087, HR = 5.32 (95 % CI = 0.62–45.6)], suggesting that capecitabine provided no additional benefit and may have deteriorated RFS. On the contrary, in the mt signature groups, the RFS of the FEC+TX group tended to be better than that of the FEC+T group (log-rank *P* = 0.065, HR = 0.44 [95 % CI = 0.18–1.07]), indicating that the add-on effect of capecitabine in the mt signature group was more pronounced than in the wt signature group. A recent study reported that the addition of postoperative chemotherapy (capecitabine or S-1) improved prognosis in cases without pCR after NAC [5,6]. Our study results suggested that additional postoperative chemotherapy is considerably beneficial to the *TP53* mt signature group, whereas it does not provide any benefit for the wt group. Therefore, *TP53* signatures can provide valuable information to guide the selection of perioperative chemotherapy in early-stage breast cancer.

While the *TP53* signature has demonstrated strong predictive value for pCR, other multi-gene expression assays, such as the 21-gene recurrence score (RS), the 70-gene signature and PAM50, have also been validated to predict pCR in breast cancer patients undergoing NAC. Studies have shown that patients with a high RS or high-risk are more likely to achieve pCR compared to low RS or low-risk [[33], [34], [35], [36]]. Similarly, PAM50 has demonstrated that HER2-enriched and Basal-like subtypes have higher pCR rates compared to Luminal subtypes [37]. While these multi-gene expression assays target specific subtypes of breast cancer, the *TP53* signature focuses on the mutation status and expression profiles of *TP53*-associated genes, offering a targeted approach that may provide advantages in specificity and applicability across all breast cancer subtypes.

This study has identified several key insights into the predictive value of the *TP53* signature for pCR and prognosis in breast cancer patients undergoing NAC. However, there are several knowledge gaps and limitations that need to be addressed to further advance this research. First, the cohorts used in this study comprised exclusively Japanese patients, which may limit the generalizability of our findings to other racial and ethnic populations. Previously, we reported on the relationship between the efficacy of NAC and *TP53* signature using multiple breast cancer cohorts consisting of patients from Western countries [26]. Given that the findings regarding the pCR rate aligned with the previous report, the clinical significance of the *TP53* signature is presumed to be consistent between Japanese and Western populations. However, further studies involving more diverse populations are needed to confirm these findings. Second, we used QpCR as our definition of pCR. This was because the results of the pooled analysis of the JBCRG studies showed that QpCR was most associated with prognosis than other definitions of pCR [29,30]. However, a meta-analysis of pCR definitions showed that ypT0ypN0 was most associated with prognosis [4]. In this study, defining ypT0ypN0 as pCR was difficult because of the limited number of cases in which ypT0ypN0 was obtained. Although not shown as a result, the ypT0ypN0 pCR rate was significantly higher in the mt signature group than in the wt signature group (OR = 9.862, 95 % CI = 2.347–41.442). Future studies should aim to include a larger number of cases to validate the findings using different pCR definitions. Third, the PC-naïve_HrR+ cohort was retrospectively collected since prospective cohorts with identical features were unavailable. To minimize selection bias, we enrolled consecutive cases from each institution; however, retrospective data collection carries inherent limitations. Prospective studies are needed to confirm these findings and to reduce the potential biases associated with retrospective data. Lastly, the study was limited by the sample size of certain subgroups, which may affect the reliability of the results. Smaller sample sizes can lead to reduced statistical power and may not accurately represent the broader population. Future research should involve larger cohorts to ensure more robust and generalizable findings.

## Conclusion

We developed a *TP53* signature diagnostic system and confirmed its ability to predict pCR and prognosis. Furthermore, our results suggest that NAC effectively improves prognosis in the *TP53* mt signature group, while the effect is limited in the wt signature group. Our results also suggest that the addition of capecitabine to FEC+T as NAC effectively improves prognosis in the mt signature group but provides limited benefits in the wt signature group. The *TP53* signature can provide valuable information for optimizing perioperative treatment strategies in breast cancer.

## Availability of data and materials

The datasets used in the current study are available from the corresponding author on reasonable request.

## CRediT authorship contribution statement

**Shin Takahashi:** Writing – review & editing, Writing – original draft, Visualization, Validation, Resources, Methodology, Investigation, Funding acquisition, Formal analysis, Data curation, Conceptualization. **Nobuaki Sato:** Writing – review & editing, Resources. **Kouji Kaneko:** Writing – review & editing, Resources. **Norikazu Masuda:** Writing – review & editing, Resources. **Masaaki Kawai:** Writing – review & editing, Resources. **Hisashi Hirakawa:** Writing – review & editing, Resources. **Tadashi Nomizu:** Writing – review & editing, Resources. **Hiroji Iwata:** Writing – review & editing, Resources. **Ai Ueda:** Writing – review & editing, Resources. **Takashi Ishikawa:** Writing – review & editing, Resources. **Hiroko Bando:** Writing – review & editing, Conceptualization. **Yuka Inoue:** Writing – review & editing, Resources. **Takayuki Ueno:** Writing – review & editing, Resources. **Shinji Ohno:** Writing – review & editing, Resources. **Makoto Kubo:** Writing – review & editing, Resources. **Hideko Yamauchi:** Writing – review & editing, Resources. **Masahiro Okamoto:** Writing – review & editing, Resources. **Eriko Tokunaga:** Writing – review & editing, Resources. **Shunji Kamigaki:** Writing – review & editing, Resources. **Kenjiro Aogi:** Writing – review & editing, Resources. **Hideaki Komatsu:** Writing – review & editing, Resources. **Masahiro Kitada:** Writing – review & editing, Methodology. **Yasuaki Uemoto:** Writing – review & editing, Methodology. **Tatsuya Toyama:** Writing – review & editing, Resources. **Yutaka Yamamoto:** Writing – review & editing, Resources. **Toshinari Yamashita:** Writing – review & editing, Resources. **Takehiro Yanagawa:** Writing – review & editing, Resources. **Hiroko Yamashita:** Writing – review & editing, Resources. **Yoshiaki Matsumoto:** Writing – review & editing, Resources. **Masakazu Toi:** Writing – review & editing, Resources. **Minoru Miyashita:** Writing – review & editing, Resources. **Takanori Ishida:** Writing – review & editing, Resources. **Fumiyoshi Fujishima:** Writing – review & editing, Formal analysis. **Satoko Sato:** Writing – review & editing, Formal analysis. **Takuhiro Yamaguchi:** Writing – review & editing, Methodology, Data curation. **Fumiaki Takahashi:** Writing – review & editing, Visualization, Validation, Methodology, Investigation, Formal analysis, Data curation. **Chikashi Ishioka:** Writing – review & editing, Supervision, Project administration, Methodology, Funding acquisition, Conceptualization.

## Declaration of competing interest

The authors declare the following financial interests/personal relationships which may be considered as potential competing interests:

ST reports honoraria from Ono, Chugai, Yakult Honsha, Taiho, Asahi Kasei Pharma, Eisai, Bristol-Myers Squibb and Eli Lilly; Prof. NM reports grants from Chugai, Eli Lilly, Astra Zeneca, Pfizer, Daiichi-Sankyo, MSD, Eisai, Novartis, Sanofi, Kyowa-Kirin, Nippon-Kayaku, Ono and Gilead Science, and honoraria from Chugai, Pfizer, Astra Zeneca, Eli Lilly and Daiichi-Sankyo, and Representative of a board of directors of JBCRG and Board of Directors of JBCS; MK reports honoraria from Pfizer, Daiichi-Sankyo, Astra Zeneca, Eli Lilly, Taiho, Kyowa-Kirin and Chugai; HI reports grants from Chugai, Daiichi-Sankyo and Astra Zeneca, and consulting fee from Daiichi-Sankyo, Chugai, Astra Zeneca, Eli Lilly, MSD, Pfizer and Gilead Science, and honoraria from Daiichi-Sankyo, Chugai, Astra Zeneca, Eli Lilly, MSD, Pfizer, Taiho and Kyowa-Kirin; TI reports honoraria from Pfizer, Kyowa-Kirin, Chugai, Daiichi-Sankyo, Astra Zeneca and Eli Lilly; TU reports grant from Eli Lilly, and honoraria from Chugai, Eisai, Astra Zeneca and Novartis; SO reports honoraria from Chugai, MSD, Nippon-Kayaku and Eli Lilly; HY reports grants from Eli Lilly and Eiken Kagaku; ET reports honoraria from Daiichi-Sankyo, Eli Lilly and Astra Zeneca; KA reports grants from Chugai, Eisai and Takeda, and consulting fee from Taiho, and honoraria from Chugai, Eisai, Astra Zeneca, Taiho, Novartis, Daiichi-Sankyo, Mochida, Ono, Pfizer, Eli Lilly, Terumo and Becton Dickinson; TT reports grants from Eli Lilly, Chugai, Eisai, Takeda, Kyowa-Kirin, Daiichi-Sankyo and Nippon-Kayaku, and honoraria from Daiichi-Sankyo, Pfizer, Novartis, Astra Zeneca, Chugai and Takeda; YY reports grants from Chugai, Kyowa-Kirin, Eisai, Daiichi-Sankyo, Nippon-Kayaku, Taiho, Takeda, Eli Lilly, Pfizer and Novartis, and honoraria from Astra Zeneca, Chugai, Kyowa-Kirin, Novartis, Eli Lilly, Pfizer, Daiichi-Sankyo, Nippon-Kayaku, Taiho, Eisai, Takeda, MSD, Sysmex and Exact Science, and participation on Advisory Board of Astra Zeneca, Chugai, Novartis, MSD, Eli Lilly, Pfizer and Daiichi-Sankyo, and member of the Board of Directors of JBCS and JBCRG; TY reports grants from Chugai, Taiho, Nippon-Kayaku and Kyowa-Kirin, and honoraria from Chugai, Eisai, Novartis, Taiho, Nippon-Kayaku, Astra Zeneca, Kyowa-Kirin, Pfizer, Eli Lilly and Daiichi-Sankyo; TY reports honoraria from Pfizer, Eli Lilly and Eisai; MT reports grants from Chugai, Takeda, Pfizer, JBCRG assoc., KBCRN assoc., Eisai, Eli Lilly, Daiichi-Sankyo, Astra Zeneca, Astellas, Shimadzu, Yakult, Nippon-Kayaku, AFI technology, Luxonus, Shionogi, GL Science and Sanwa Shurui, and honoraria from Chugai, Takeda, Pfizer, Kyowa-Kirin, Taiho, Eisai, Daiichi-Sankyo, Astra Zeneca, Eli Lilly, MSD, Exact Science, Novartis, Shimadzu, Yakult, Nippon-Kayaku, Devicore Medical Japan and Sysmex, and advisory board of Daiichi-Sankyo, Eli Lilly, BMS, Athenex Oncology, Bertis Terumo and Kansai Medical Net, and a member of the board of directors of JBCRG, KBCRN and OOTR, and chairman of the board of directors of JBCS, and associate editor of British Journal of Cancer, Scientific Reports, Breast Cancer Research and Treatment, Cancer Science, Frontiers in Women's Cancer, Asian Journal of Surgery and Asian Journal of Breast Surgery; TY reports grants from AC Medical, A2 Healthcare, ClinChoice, Japan Tabacco, Japan Media, Medidata Solutions, Ono, Kyowa-Kirin, Tsumura, Daiichi-Sankyo, Otsuka, Eisai, Solasia Pharma, Asahi Intecc, 3H Clinical Trial, Medrio, Nipro, Intellim, Welby, 3H Medi Solution, Baseconnect, Nobori, Puravida Technologies and Hemp Kitchen, and consulting fee from Public Health Research Foundation, EPS, Japan Tabacco, Medidata Solutions, Ono, Kowa, Chugai, Daiichi-Sankyo, Eisai, 3H Clinical Trial, Intellim, Astra Zeneca, Sonire Therapeutics, Seikagaku, Merck, Mebix and Nippon Boehringer Ingelheim, and advisory board of Incyte Bioscience; CI reports grants from Chugai, Asahi Kasei Pharma, Ono, Eli Lilly, Shionogi, Yakult, Otsuka, Sanofi, Merck, Astra Zeneca, Nippon Boehringer Ingelheim, Yansen, MSD, Sonia-Therapy, Taiho, Takeda, Daiichi-Sankyo, Bayer, Nippon-Kayaku, Tsumura, Kyowa-Kirin, Hitachi, Incyte, Ascent, Eisai, PRA Health Science, Novartis and Riken Genesis, and honoraria from Taiho, Merck, Eli Lilly, Bristol-Myers Squibb, Daiichi-Sankyo, Nippon-Kayaku, Nihon-Servier, M3, MSD, Novartis, Astra Zeneca, Ono, Sanofi, Chugai, Terumo, Asahi Kasei Pharma, Pfizer, Bayer, Incyte, Takeda and Kyowa-Kirin. All remaining authors declare no competing interests.

## Appendix Supplementary materials

Supplemental Figure 1. Hierarchical cluster (A) and receiver operating characteristic (ROC) (B) analyses of the HG/MCC cohort in the development cohorts Image, application 1

Supplemental Figure 2. RFS of all cases and the subgroups of the HG/MCC cohort Image, application 2

Supplemental Figure 3. RFS and OS of the subgroups of the NAC cohort Image, application 3

Supplemental Table 1. Gene set of *TP53* signature diagnostic system Image, application 4

Supplemental Table 2. Relationship of each *TP53* status determined by *TP53* signature diagnostic system and direct sequencing in TUH cohort Image, application 5

Supplemental Table 3. Patient clinicopathological background disaggregated by *TP53* signature status in HG/MCC cohort Image, application 6

Supplemental Table 4. Stratified analysis of pCR rate by ER and PgR Image, application 7

Supplemental Table 5. Logistic regression analysis for pCR ratio Image, application 8

Supplemental Table 6. Patient clinicopathological background disaggregated by *TP53* signature status in NAC (HR+/HER2−) cohort and PC-naïve (HR+/HER2−) cohort Image, application 9

Supplemental Table 7. Patient clinicopathological background disaggregated by *TP53* signature status in OOTR-N003 cohort Image, application 10

Image, application 11
