# Supplementary material for: TP53 signature predicts pathological complete response after neoadjuvant chemotherapy for breast cancer: Observational and confirmational study using prospective study cohorts
Source: Transl Oncol. 2024 Jul 24;48:102060. doi: 10.1016/j.tranon.2024.102060 (PMC11325231; doi:10.1016/j.tranon.2024.102060)
Supplement: Supplementary file 1 — Supplemental Figure 1. Hierarchical cluster (A) and receiver operating characteristic (ROC) (B) analyses of the HG/MCC cohort in the development cohorts [file mmc1.pdf]

Supplemental Fig. 1

A

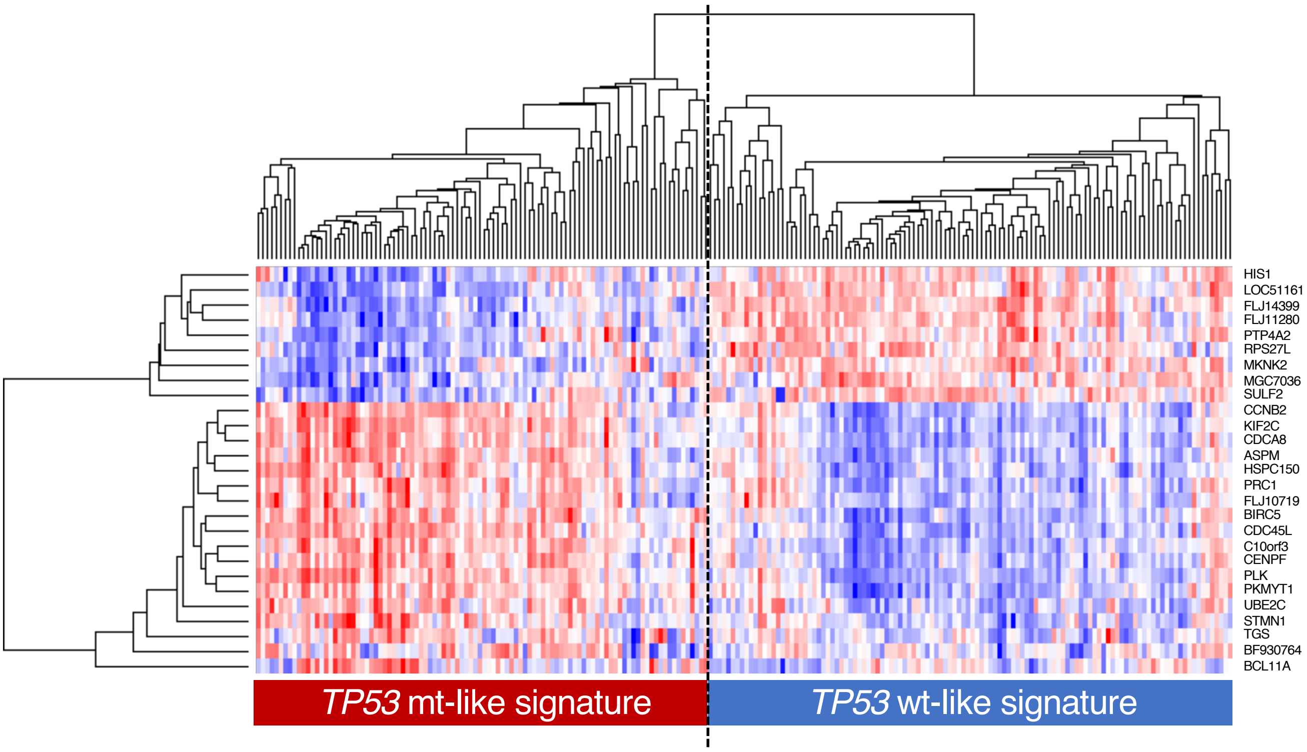

B

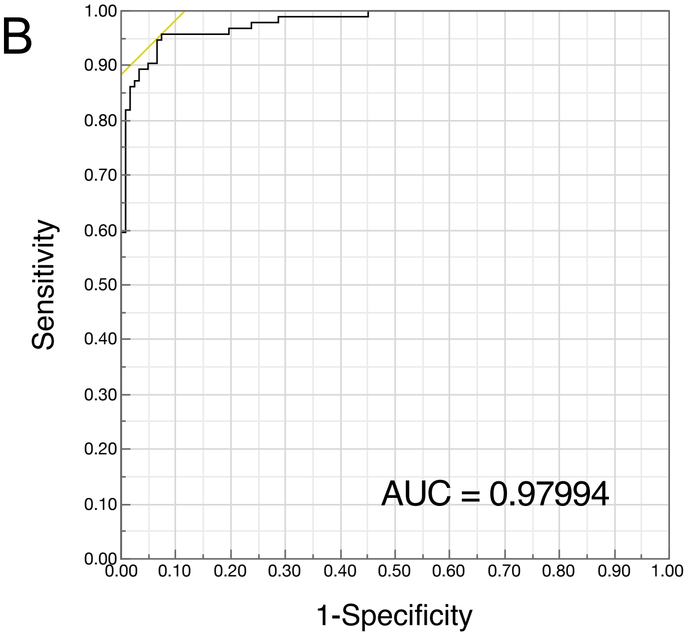

Hierarchical cluster (A) and receiver operating characteristic (ROC) (B) analyses of the HG/MCC cohort in the development cohorts
