# Supplementary material for: TP53 signature predicts pathological complete response after neoadjuvant chemotherapy for breast cancer: Observational and confirmational study using prospective study cohorts
Source: Transl Oncol. 2024 Jul 24;48:102060. doi: 10.1016/j.tranon.2024.102060 (PMC11325231; doi:10.1016/j.tranon.2024.102060)
Supplement: Supplementary file 2 — Supplemental Figure 2. RFS of all cases and the subgroups of the HG/MCC cohort [file mmc2.pdf]

Supplemental Fig. 2

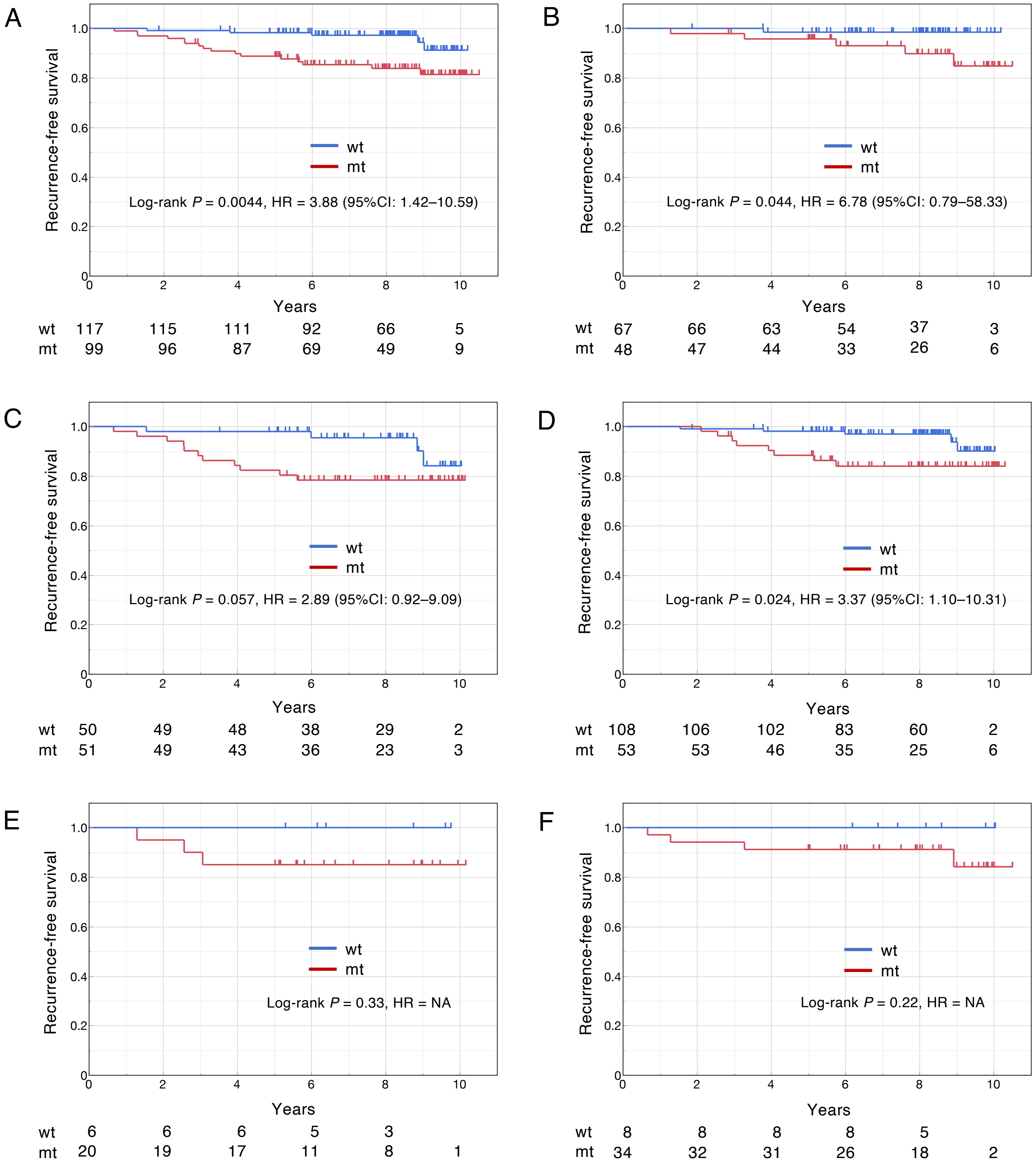

RFS of all cases and the subgroups of the HG/MCC cohort

A, All cases; B, pStage I subgroup; C, pStage II subgroup; D, ER-positive subgroup; E, HER2-positive subgroup; E, TNBC subgroup

RFS, recurrence-free survival; ER, estrogen receptor; HER2, human epidermal growth factor receptor type 2; TNBC, triple-negative breast cancer; wt, wild-type signature, mt, mutant signature; HR, hazard ratio; CI: confidence interval.
