# Supplementary material for: TP53 signature predicts pathological complete response after neoadjuvant chemotherapy for breast cancer: Observational and confirmational study using prospective study cohorts
Source: Transl Oncol. 2024 Jul 24;48:102060. doi: 10.1016/j.tranon.2024.102060 (PMC11325231; doi:10.1016/j.tranon.2024.102060)
Supplement: Supplementary file 3 — Supplemental Figure 3. RFS and OS of the subgroups of the NAC cohort [file mmc3.pdf]

Supplemental Fig. 3

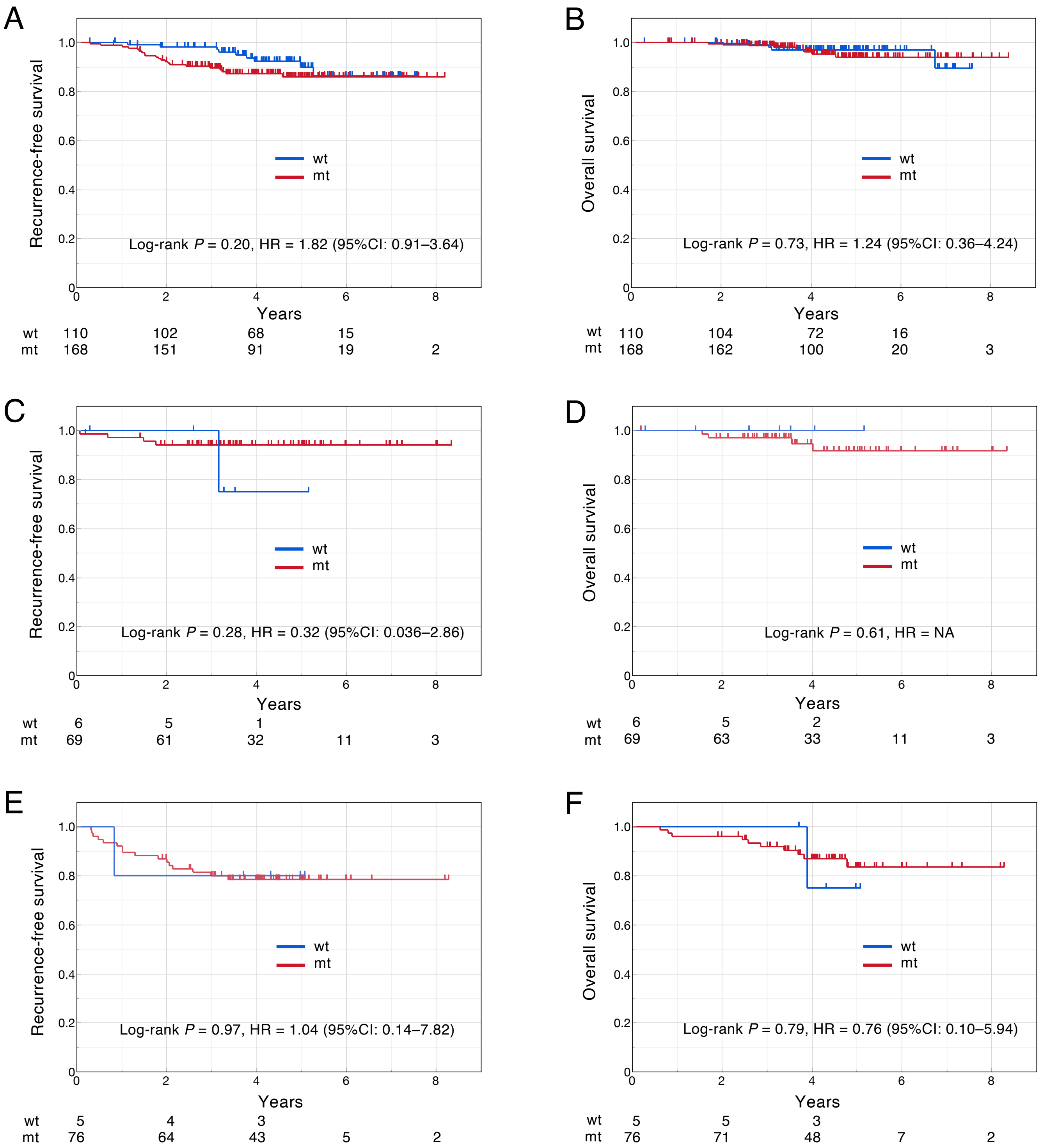

RFS and OS of the subgroups of the NAC cohort

RFS (A) and OS (B) of HrR-positive subgroup; RFS (C) and OS (D) of HER2-positive subgroup; RFS (E) and OS (F) of TNBC subgroup

RFS, recurrence-free survival; OS, overall survival; HrR, hormone receptor; HER2, human epidermal growth factor receptor type 2; TNBC, triple-negative breast cancer; wt, wild-type signature, mt, mutant signature; HR, hazard ratio; CI: confidence interval.
