# Supplementary material for: TP53 signature predicts pathological complete response after neoadjuvant chemotherapy for breast cancer: Observational and confirmational study using prospective study cohorts
Source: Transl Oncol. 2024 Jul 24;48:102060. doi: 10.1016/j.tranon.2024.102060 (PMC11325231; doi:10.1016/j.tranon.2024.102060)
Supplement: Supplementary file 11 [file mmc11.docx]

***TP53* signature predicts pathological complete response after neoadjuvant chemotherapy for breast cancer: observational and confirmational study using prospective study cohorts**

Supplementary Information

**Supplemental methods**

**Details of cohorts**

The development cohorts

The Tohoku University Hospital (TUH) cohort (n = 37): This cohort consists of primary breast cancer patients who underwent surgery at TUH. Patients were selected based on the availability of surgical specimens and the presence of *TP53* gene mutation data, which were obtained via direct sequencing in our previous study^1^. From the original TUH cohort (n = 40), only patients whose specimens were available were included in the analysis.

The Hoshi General Hospital/Miyagi Cancer Center (HG/MCC) cohort (n = 216): This cohort comes from a prospective observational study of primary breast cancer patients who received surgery between February 2008 and February 2014 in Hoshi general hospital or Miyagi Cancer Center ^2,3^. From the original HG/MCC cohort including stage 0–IV patients (n = 260), only Stage I–II (which is relatively close to the selection criteria of the NAC cohort [cT1c-3 cN0 cM0 (>1cm) / cT1-3 cN1 cM0] patients whose specimens were available were included in the analysis.

The TUH cohort and HG/MCC cohort were used to develop the *TP53* signature diagnostic kit and determine the cut-off value for diagnosing *TP53* signature status. The HG/MCC cohort was used to evaluate the prognostic value of the *TP53* signature.

The validation cohorts

NAC cohort (n = 407): This cohort consists of patients from 5 prospective clinical studies (JBCRG-01, JBCRG-02, JBCRG-02', JBCRG-03, and OOTR-N003) in which NAC was administered to primary breast cancer patients with cT1c-3 cN0 cM0 (>1cm) / cT1-3 cN1 cM0 ^4-8^. Patients whose specimens were available were included in the analysis. Patients in the JBCRG-01, -02, and -02’ studies received NAC comprising 4 cycles of FEC (fluorouracil 500 mg/m^2^, epirubicin 100 mg/m^2^, and cyclophosphamide 500 mg/m^2^) on day 1 every 21 days followed by 4 cycles of docetaxel (75 mg/m^2^) on day 1 every 21 days. Patients in the JBCRG-03 study received NAC comprising 4 cycles of docetaxel (75 mg/m^2^) followed by 4 cycles of FEC. The OOTR-N003 study compared 4 cycles of FEC followed by 4 cycles of docetaxel (75 mg/m^2^) with 4 cycles of docetaxel (75 mg/m^2^) + capecitabine (825 mg/m^2^ twice daily on days 1–14) as NAC.

Perioperative chemotherapy (PC)-naïve, hormone receptor-positive (positive for both or one of ER and PgR) breast cancer cohort (PC-naïve_HrR+ cohort) (n = 322): This retrospectively collected cohort consists of HrR+ breast cancer patients (cT1c-3 cN0 cM0 (>1 cm) / cT1-3 cN1 cM0; which is same as the patient selection criteria of the NAC cohort) who underwent surgery without PC between August 2005 and July 2009 for matching the enrollment period of the NAC cohort. To eliminate case selection bias, we enrolled up to 60 consecutive eligible patients per institution from August 1, 2005.

The NAC cohort was used to test whether the *TP53* signature diagnosis kit could predict pCR after NAC. The PC-naïve_HrR+ cohort was compared with the HrR+ subgroup of the NAC cohort to test whether there were differences in the prognostic significance of NAC by *TP53* signature status.

Specimens were collected and stored in accordance with standard protocols to ensure sample integrity.

***TP53* signature diagnostic kit**

Oligonucleotide probes for gene expression analysis on the nCounter (Nanostring, Seattle, WA, USA) platform were synthesized for a total of 33 genes, which comprised the *TP53* signature, as well as five internal control genes (*RPLP0*, *PSMC4*, *TBP*, *GAPDH*, and *TFRC*). These probes were designed based on the sequences of the target genes by NanoString. Six of the genes (*ANAPC*, *CENPE*, *MAPRE1*, *MGC45866*, *MUTYH*, and *PTTG1*) were excluded due to insufficient expression levels in preliminary analysis. Finally, the *TP53* signature gene set was created with a total of 32 genes, including 27 *TP53* signature genes (18 upregulated and 9 downregulated genes in patients with breast cancer with mutant *TP53* gene) and 5 internal control genes (Supplemental Table 1). The nCounter platform uses a digital barcoding technology that allows for direct multiplexed measurement of gene expression with high precision and sensitivity. Quality control measures, such as the use of internal control genes and technical replicates, were implemented to ensure the accuracy and reliability of the gene expression data.

**Method for diagnosis of *TP53* signature status**

The raw expression levels of the 27 genes were converted to natural logarithms. The ratio of the sum of logarithmic expression levels of 18 upregulated genes to the sum of logarithmic expression levels of 9 downregulated genes was used to define the *TP53* signature score. If the *TP53* signature score of a certain sample was equal to or greater than the cut-off value, the sample was labeled as having a *TP53* mt signature; otherwise, it was labeled as having a *TP53* wt signature.

The cut-off value for the *TP53* signature score was determined using the TUH and HG/MCC cohorts. The *TP53* signature expression data of the HG/MCC cohort were standardized using nSolver (Nanostring, Seattle, WA, USA). Next, unsupervised hierarchical cluster analysis was performed using the standardized expression data to classify the samples into *TP53* signature mt- and wt-like groups. The hierarchical clustering was performed using the average linkage clustering method with Pearson correaltion as the measure of similarity. The optimal cut-off *TP53* signature score for classification into mt- and wt-like groups was determined using receiver operating characteristic (ROC) analysis.

Then, with the TUH cohort, the concordance rate between the *TP53* signature status, determined using the diagnostic kit, and the *TP53* gene mutation status, determined via direct sequencing, was examined. The concordance rate was calculated as the percentage of samples with matching *TP53* signature status and *TP53* gene mutation status.

**Specimens and RNA extraction**

Regarding the NAC cohort, formalin-fixed, paraffin-embedded (FFPE) specimens from biopsies performed before NAC were used. In the case of the remaining three cohorts, either FFPE biopsy specimens or surgical specimens were used. Tumor cells were collected from unstained FFPE tissues via macrodissection, with reference to the hematoxylin–eosin stained specimen. Total RNA was extracted using the RNeasy FFPE kit (Qiagen, Valencia, CA, USA) or the Maxwell RSC RNA FFPE Kit (Promega, Fitchburg, USA). Patients with <100 ng of total RNA obtained were excluded from the analysis (insufficient tissue, Fig. 1). The maximum amount of RNA used for analysis was 300 ng, and if the amount of total RNA obtained was <300 ng, the entire amount was used for analysis. Extracted RNA was stored at -80°C until further use to ensure its stability and integrity.

**Clinicopathological factors**

Clinicopathological background factors were collected, including age, menopausal status, T stage, N stage, estrogen receptor (ER) status, progesterone receptor (PgR) status, HER2 status, nuclear grade, and Ki-67 data. In the OOTR-N003 cohort, all data were obtained from clinical trial data ^8^. In the HG/MCC and JBCRG cohorts, data other than nuclear grade and Ki-67 were collected from clinical trial data ^2-7^. In the TUH and PC-naïve_HrR+ cohorts, data other than nuclear grade and Ki-67 were collected from medical records. Clinicopathological data were extracted from patients’ medical records and pathology reports to ensure comprehensive data collection. Nuclear grade and Ki-67 data in the HG/MCC, JBCRG, and PC-naïve_HrR+ cohorts were obtained by the Department of Pathology, TUH. Immunohistochemical staining for Ki-67 was performed using Autostainer Link 48 (Agilent Technologies, Santa Clara, CA, USA) according to recommended procedures.

**Supplementary Results**

**Cut-off value for *TP53* signature diagnostic system**

The *TP53* signature gene expression data for the HG/MCC cohort (216 cases) and the TUH cohort (37 cases) were obtained using the nCounter *TP53* signature diagnostic system.

In the HG/MCC cohort, the unsupervised hierarchical clustering results revealed that 99 cases had mt- and 117 cases had wt-like *TP53* signature (Supplemental Fig. 1A). The *TP53* signature scores were calculated for each case and the optimal cut-off value of the *TP53* signature score for classifying cases into mt-like and wt-like breast cancer was determined to be 1.67 (area under the ROC curve = 0.97994) using ROC analysis (Supplemental Fig. 1B). The *TP53* signature status of the cases in the TUH cohort was determined using the abovementioned cut-off value, and 16 and 21 cases were classified as having mt and wt signatures, respectively. The concordance rate of the *TP53* signature status determined using nCounter and the *TP53* gene mutation status determined using direct sequencing was 94.6% (35/37) (Supplemental Table2). These results indicate that the diagnostic ability of *TP53* signature with 27 genes for *TP53* mutation status is comparable to that of original *TP53* signature with 33 genes.

**Details about comparisons of patient clinicopathological background in HG/MCC cohort**

The *TP53* mt signature group had significantly more ER-negative, PgR-negative, HER2-positive, higher grade, higher Ki-67 cases, and cases who underwent adjuvant chemotherapy and adjuvant endocrine therapy than the wt signature group.

**Details about comparisons of patient clinicopathological background in NAC (HrR+/HER2−) cohort and PC-naïve (HrR+/HER2−) cohort**

Comparisons of patient clinicopathological background factors disaggregated by *TP53* signature status in NAC (HrR+/HER2−) cohort and PC-naïve (HrR+/HER2−) cohort were shown in Supplemental table 6. In the NAC (HrR+/HER2−) group, patients with younger age, premenopausal, higher T stage, N1, ER-negative, PgR-negative, and high-grade diseases were significantly more prevalent than in the PC-naïve (HrR+/HER2−) group, indicating that the former had a higher proportion of patients with poor prognostic factors than the latter. In the NAC (HrR+/HER2−) cohort, the mt signature group [NAC (HrR+/HER2−)/mt] had significantly more cases with high grade and Ki-67 than the wt signature group [NAC (HrR+/HER2−)/wt]. In the PC-naïve (HrR+/HER2−) cohort, the mt signature group [PC-naïve (HrR+/HER2−)/mt] had significantly more PgR-negative, high grade, and high Ki-67 cases. Between the NAC (HrR+/HER2−)/wt and PC-naïve (HrR+/HER2−)/wt groups and between the NAC (HrR+/HER2−)/mt and PC-naïve (HrR+/HER2−)/mt groups, the former groups had significantly more patients who were relatively young, premenopausal, high T stage, N1, and high-grade diseases compared with the latter groups. No difference in the proportion of cases with high Ki-67 was observed between the NAC (HrR+/HER2−)/wt and the PC-naïve (HrR+/HER2−)/wt groups. In contrast, the proportion of cases with high Ki-67 in the PC-naïve (HrR+/HER2−)/mt group was significantly higher than that in the NAC (HrR+/HER2−)/mt group.

**Details about comparisons of patient clinicopathological background in the OOTR-N003 cohort**

The comparison of patient clinicopathological background factors (Supplemental Table 7) showed no significant intergroup differences except in terms of T stage; the FEC+TX group had significantly fewer high T cases than the FEC+T group. In the FEC+T group, the mt signature (FEC+T/mt) group had significantly more cases with high T, ER-negative, PgR-negative, HER2-positive, high-grade, and high Ki-67 than the wt signature (FEC+T/wt) group. In the FEC+TX group, the mt signature (FEC+TX/mt) group had significantly more cases with ER-negative, PgR-negative, high-grade, and high Ki-67 than the wt signature (FEC+TX/wt) group. The FEC+T/wt group had significantly more cases with PgR-negative cases and high Ki-67 than the FEC+TX/wt group. The proportion of high T cases in the FEC+T/mt group was significantly larger than that in the FEC+TX/mt group.

**Reference**

1 Takahashi, S. *et al.* Prediction of breast cancer prognosis by gene expression profile of TP53 status. *Cancer Sci* **99**, 324-332 (2008). <https://doi.org:10.1111/j.1349-7006.2007.00691.x>

2 Yamaguchi, S. *et al.* Molecular and clinical features of the TP53 signature gene expression profile in early-stage breast cancer. *Oncotarget* **9**, 14193-14206 (2018). <https://doi.org:10.18632/oncotarget.24447>

3 Takahashi, S. *et al.* TP53 signature diagnostic system using multiplex reverse transcription-polymerase chain reaction system enables prediction of prognosis of breast cancer patients. *Breast Cancer* **28**, 1225-1234 (2021). <https://doi.org:10.1007/s12282-021-01250-z>

4 Toi, M. *et al.* Phase II study of preoperative sequential FEC and docetaxel predicts of pathological response and disease free survival. *Breast Cancer Res Treat* **110**, 531-539 (2008). <https://doi.org:10.1007/s10549-007-9744-z>

5 Iwata, H. *et al.* Docetaxel followed by fluorouracil/epirubicin/cyclophosphamide as neoadjuvant chemotherapy for patients with primary breast cancer. *Jpn J Clin Oncol* **41**, 867-875 (2011). <https://doi.org:10.1093/jjco/hyr081>

6 Kuroi, K. *et al.* Comparison of different definitions of pathologic complete response in operable breast cancer: a pooled analysis of three prospective neoadjuvant studies of JBCRG. *Breast Cancer* **22**, 586-595 (2015). <https://doi.org:10.1007/s12282-014-0524-4>

7 Kuroi, K. *et al.* Prognostic significance of subtype and pathologic response in operable breast cancer; a pooled analysis of prospective neoadjuvant studies of JBCRG. *Breast Cancer* **22**, 486-495 (2015). <https://doi.org:10.1007/s12282-013-0511-1>

8 Ohno, S. *et al.* Randomized trial of preoperative docetaxel with or without capecitabine after 4 cycles of 5-fluorouracil- epirubicin-cyclophosphamide (FEC) in early-stage breast cancer: exploratory analyses identify Ki67 as a predictive biomarker for response to neoadjuvant chemotherapy. *Breast Cancer Res Treat* **142**, 69-80 (2013). <https://doi.org:10.1007/s10549-013-2691-y>
